# 3D Printing of Thermoresponsive Polyisocyanide (PIC) Hydrogels as Bioink and Fugitive Material for Tissue Engineering

**DOI:** 10.3390/polym10050555

**Published:** 2018-05-21

**Authors:** Nehar Celikkin, Joan Simó Padial, Marco Costantini, Hans Hendrikse, Rebecca Cohn, Christopher J. Wilson, Alan Edward Rowan, Wojciech Święszkowski

**Affiliations:** 1 Faculty of Materials Science and Engineering, Warsaw University of Technology, 141 Woloska str., 02-507 Warsaw, Poland; nehar.celikkin@pw.edu.pl (N.C.); marco.costantini@uniroma1.it (M.C.); hc.hendrikse@gmail.com (H.H.); cohnr@umich.edu (R.C.); 2 Department of Molecular Materials, Radboud Universities, Heyendaalseweg 135, 6525 AJ Nijmegen, The Netherlands; joansimopadial@gmail.com (J.S.P.); a.rowan@science.ru.nl (A.E.R.); 3 Noviotech B.V., Molenveldlaan 43, 6523 RJ Nijmegen, The Netherlands; c.wilson@noviosense.com

**Keywords:** thermoresponsive polymers, 3D hydrogel scaffolds, 3D bioprinting, polyisocyanide (PIC) hydrogels, dual hydrogel system

## Abstract

Despite the rapid and great developments in the field of 3D hydrogel printing, a major ongoing challenge is represented by the development of new processable materials that can be effectively used for bioink formulation. In this work, we present an approach to 3D deposit, a new class of fully-synthetic, biocompatible PolyIsoCyanide (PIC) hydrogels that exhibit a reverse gelation temperature close to physiological conditions (37 °C). Being fully-synthetic, PIC hydrogels are particularly attractive for tissue engineering, as their properties—such as hydrogel stiffness, polymer solubility, and gelation kinetics—can be precisely tailored according to process requirements. Here, for the first time, we demonstrate the feasibility of both 3D printing PIC hydrogels and of creating dual PIC-Gelatin MethAcrylate (GelMA) hydrogel systems. Furthermore, we propose the use of PIC as fugitive hydrogel to template structures within GelMA hydrogels. The presented approach represents a robust and valid alternative to other commercial thermosensitive systems—such as those based on Pluronic F127—for the fabrication of 3D hydrogels through additive manufacturing technologies to be used as advanced platforms in tissue engineering.

## 1. Introduction

Over the past few decades, due to increased life expectancy and the limited number of healthy donors, Tissue Engineering (TE) has emerged to create patient-specific innovative solutions [1]. The necessity of growing cells in actual 3D matrices has driven multidisciplinary research to develop both innovative biocompatible materials and technologies to process them. In particular, the recent development of rapid prototyping techniques and their application in the biofabrication research field has demonstrated great potential towards this goal [2,3].

Nowadays, additive manufacturing techniques represent a fast and cost-effective technology, able to create 3D objects with high precision, resolution, and repeatability [4,5]. Thanks to these attractive features, 3D printing and bioprinting are rapidly becoming a first-choice approach for the production of engineered materials for tissue engineering. Besides the aforementioned advantages, these innovative techniques are superior to other biofabrication techniques, as they allow to create highly customized intricate architectures with a broad range of materials, including thermoplastic polymers [6,7], metals and alloys [8,9], ceramics [10,11], and hydrogels [12,13,14,15]. Among these materials, hydrogels have gained the greatest attention, as they represent an ideal biomimetic environment where cells can migrate and self-organize to form a functional micro or mini tissue. However, a number of design criteria—such as hydrogel stiffness, cross-linking density, the quantity of adhesion moieties, etc.—should be tailored to favor the formation of a functional neo-tissue. In fact, it is well-known that the physicochemical, mechanical, and biological properties of the matrix in which cells are embedded play a crucial role for a successful organization, differentiation, and maturation of the engineered tissue [16]. Thus, several research groups have been focused on studying the formulation and rheological properties of precursor hydrogel solutions (i.e., *bioinks*), as well as to the synthesis of new polymers with improved hydrogel performances.

Recently, reverse thermoresponsive hydrogels—such as those based on Pluronic F127—have been processed through a 3D printing technique. However, pristine Pluronic F127 hydrogel structures cannot support cells in the long term, due to their high density, lack of adhesive moieties, and insufficient nutrient transport [17,18]. Therefore, they have mostly been employed as fugitive [19], support materials [20] or deposited in combination with other biomaterials that can be crosslinked in a later step or form a stable gel simultaneously to their deposition [19,21].

An innovative and fully synthetic PolyIsoCyanide (PIC) thermoresponsive material, capable of mimicking characteristics of the natural ExtraCellular Matrix (ECM), has recently been reported by Das et al. [22]. This material exhibits a reverse thermoresponsive behavior due to the hydrophobic interactions of the oligoglycol substituent present along its backbone, with a steep increase of the storage modulus (*G*′) above 18 °C. Moreover, they demonstrated that the mechanical properties and network characteristics of the resulting hydrogel can be precisely controlled in a rather broad range [23,24,25]. Furthermore, this new class of material exhibits cytocompatibility and unique reverse thermoresponsive properties [22], representing an ideal candidate for bioink formulation to be used for the assembly of 3D matrices via printing and bioprinting technologies.

In this work, we first demonstrate that PIC solutions can be deposited in 3D with good accuracy, thanks to their rheological temperature-dependent properties. We performed a thorough optimization of the 3D printing process, showing the possibility of fabricating 3D objects with a good shape fidelity. Then, we present an innovative dual hydrogel system with improved biochemical features, highly suited for advanced tissue engineering applications. In this application, we use PIC (inner gel) to create 3D structures that serve as a template within the second gel, namely Gelatin MethAcrylate (GelMA), a broadly used biopolymer that (1) can undergo a fast and cell-friendly UV crosslinking, (2) highly favors cell adhesion, spreading and differentiation of multiple cell types, and (3) represents an ideal substrate for multiple TE applications [26,27,28,29].

## 2. Materials and Methods

### 2.1. Synthesis and Characterisation of PIC

A solution of catalyst Ni(ClO_4_)_2_·6H_2_O (1 mM) in toluene/ethanol (9:1) was added to a solution of 2-(2-(2-methoxyethoxy)ethoxy)ethyl-(l)alaninyl-(d)-isocyanoalanine monomer in freshly distilled toluene (50 mg/mL total concentration) with a catalyst: a monomer ratio of 1:4000 under an inert atmosphere. The reaction mixture was stirred at room temperature (20 °C) for 96 h, and FTIR (Fourier Transform Infrared) spectroscopy analysis was performed over a range of 4000–400 cm^−1^ at a resolution of 4 cm^−1^. After the FTIR results confirmed the completion of the reaction, the resulting polymer was precipitated three times from dichloromethane in diisopropyl ether and dried overnight in the air [24]. The Molecular Weight (MW) of the resulting polymer was determined by the Mark-Houwink equation:

(1) $$\left[\eta \right]=KMW^{\alpha }$$

which defines the relationship between intrinsic viscosity ([*η*]) and the molecular weight (Equation (1)) [30]. Thus, the viscosity measurements were carried out by measuring six solutions of the PIC polymer (0.1, 0.2, 0.3, 0.4, 0.5 and 0.6 mg/mL) in acetonitrile.

### 2.2. Rheological Analysis of PIC Hydrogel

The mechanical properties and the viscosity were studied with a stress-controlled rheometer (Discovery HR-1, TA Instruments, New Castle, DE, USA). The rheometer was equipped with aluminum parallel plate geometry (40 mm diameter), and the gap between the plates was set to 250 μm. In order to verify the stiffness (*G*_0_) of the hydrogels at 37 °C, rheological measurements were performed at a controlled temperature gradient (4–50 °C), with a heating rate of 2 °C/min, a constant strain of 1% and a frequency of 1 Hz.

### 2.3. 3D Printing of PIC

The PIC (50 mg) was dissolved in 10 mL of deionized water and was printed with a 3D bioplotter (EnvisionTech GmbH, Gladbeck, Germany). The general purpose dispersing tips (Nordson EFD, RI, USA) with the following dimensions: an inner diameter of 330 µm, an outer diameter of 650 µm, and a length of 3.81 cm were used for printing. Printing was performed at 0.3 bar and varying printing speeds (25–35 mm/s, x-y traveling speed). The temperature of the printing cartridge was kept between 12 and15 °C, while the temperature of the printing bed was kept between 37 and 40 °C. To print the desired structures, the layer thickness was set to 200 µm, and the distance between the strands was set to 500 µm. All printed structures were created by means of a 3D Builder software (Microsoft Corporation) and converted into G-code via Visual Machines software (EnvisionTech GmbH, Gladbeck, Germany) for the printing process.

### 2.4. Synthesis of Gelatin Methacrylate

The GelMA was synthesized as described previously [31]. Briefly, type A porcine skin gelatin (Sigma, Poznan, Warsaw) was dissolved at 10% (*w*/*v*) into Phosphate Buffered Saline PBS at 60 °C and stirred until fully dissolved. A dose of 0.8 mL of methacrylic anhydride (Sigma) per gram of gelatin was added under constant stirring. After the reaction was completed, the mixture was dialyzed against distilled water using a 12–14 kDa cut-off dialysis tubing (Spectra/Por, Rancho Dominguez, CA, USA) for 1 week at 40 °C to remove salts and methacrylic acid. The solution was lyophilized to generate a white porous foam and stored at −80 °C until further use.

### 2.5. Creating Dual Hydrogel System: PIC as Fugitive Material

The PIC (50 mg) was dissolved in 10 mL of deionized water and printed with a 3D bioplotter (EnvisionTEC, GmbH) into a 35 mm petri dish with parameters described in 2.4. Following the printing, the petri dish was filled with 5% (*w*/*v*) of GelMA solution containing 0.05% of Irgacure 2959 (37 °C). The overall scaffold was exposed to UV light (365 nm, 12.5 mW/cm^2^) for 60 s. After the GelMA was completely crosslinked, the scaffold was washed with cold PBS to remove printed PIC structure from UV crosslinked GelMA hydrogel.

## 3. Results and Discussion

### 3.1. Synthesis of PIC Polymer

**Figure 1:**
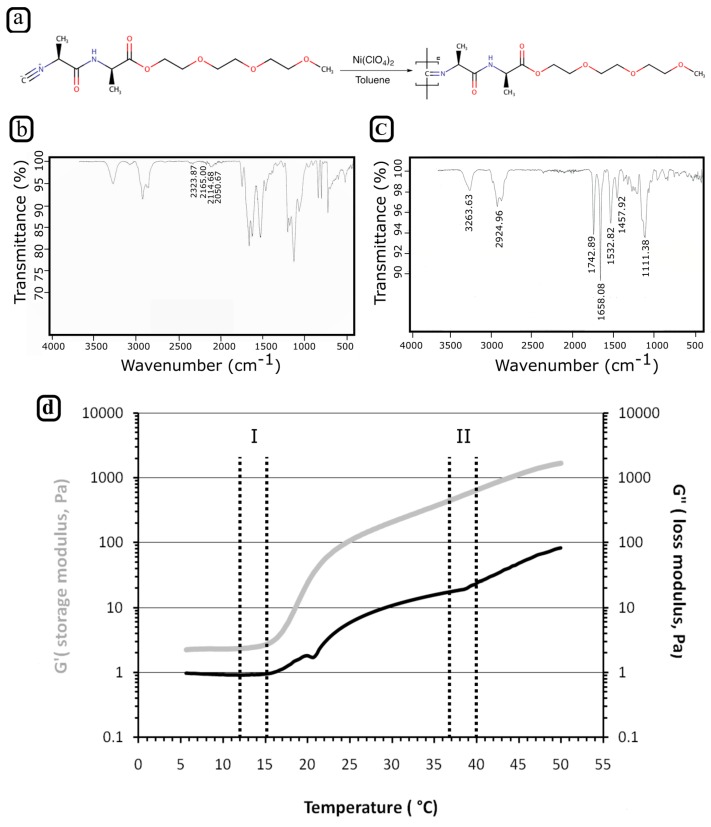
Synthesis of PIC polymer and rheological characterization of PIC hydrogels. (**a**) Chemical structure of PIC polymer, (**b**) IR-spectra of the reaction mixture, (**c**) IR-spectra of the resulting PIC polymer (**d**) Rheological data representing the loss and storage modulus as a function of temperature for 5 mg/mL PIC hydrogel solution. Dotted lines show (respectively) the ranges of (I) the cartridge temperature and (II) the printing bed temperature used in our experiments.

To use PIC effectively as a biocompatible ink, a few main criteria must be addressed. In particular, its gelation point should be below physiological temperatures (being a reverse thermoresponsive hydrogel) and its rheological properties should be suitable for 3D printing. To meet the first feature, a PIC polymer was synthesized from 2-(2-(2-methoxyethoxy)ethoxy)ethyl-(l)-alaninyl-(d)-isocyanoalanine monomers, which should give the polymer a gelation point at around 18 °C [24]. The monomer was polymerized in the presence of Ni(ClO_4_)_2_ as a catalyst (Figure 1a). The polymerization was followed by recording the peak at 2142 cm^−1^ in the IR spectra of the reaction mixture, which corresponds to the isocyanide group of the monomer (Figure 1b). By tracking this peak, the reaction progress could be followed as the isocyanide group of the monomer converted into a secondary ketamine during polymerization. After 4 days of reaction, the isocyanide group peak had completely disappeared (Figure 1c), indicating that the reaction was complete. The PIC hydrogels’ MW was determined, as the stiffness, the solubility, and the gelation characteristics of the PIC hydrogel are all important parameters strongly affected by its MW. The molecular weight was determined using the Mark-Houwink equation, which defines the relationship between viscosity and the MW. This procedure is required to measure the viscosity of solutions at different polymer concentrations. K and α given in Equation (1) were empirically determined [K = 1.4 × 10^−9^ and α = 0.5714] and are constants for the acetonitrile-PIC system. Based on the determined viscosities, K and α values of the MW were determined to be around 500 kDa.

It is worth mentioning that PIC polymers can be synthesized additionally via copolymerization of a triethylene glycol functionalized isocyano-(d)-alanyl-(l)-alanine monomer and the corresponding azide-terminal monomer to introduce cell adhesion peptides (such as GRGDS) to the polymer chain [32]. By changing the molar ratios of monomer and cellular adhesion peptides, the density of adhesion sequences can be precisely controlled, thus representing a great advantage for potential TE applications.

### 3.2. Rheology of PIC Solution

Scaffold architecture greatly influences the overall construct strength and nutrient/waste flows of cellularized scaffolds. In the case of thermoresponsive hydrogels, gelation temperature, extrusion pressure, XY plotting speed, needle type and diameter, cartridge, and printing bed temperature are the main parameters that control scaffold architecture itself. All of these parameters are closely interconnected and need optimization to ensure a reproducible cell-friendly printing process.

In particular, hydrogel gelation temperature greatly impacts the overall performance of the fiber deposition process. Hence, prior to deposition, the bioink should be in its extrudable form and, upon extrusion, it should form a stable gel strand. Therefore, we performed a rheological study of PIC solutions to evaluate precisely their gelation temperature. G’ of PIC solution is rather low for a printable hydrogel, the highest solubility of PIC in water (0.5 wt. %) was used to obtain the best possible hydrogel fibers during the printing process.

In Figure 1d, the storage modulus (G’) and the loss modulus (G”) were plotted as a function of temperature. It can be noticed that G’ and G” both increased when the temperature rises. Interestingly, we observed that the G’ trace was greater than G’’ trace for the whole temperature range explored during rheology tests, revealing that also at low temperatures the PIC solution behaves like a weak gel. Around 18 °C, the elastic modulus rapidly increased 10-fold and the difference between the two traces became more significant, indicating a sol-gel transition.

**Figure 2:**
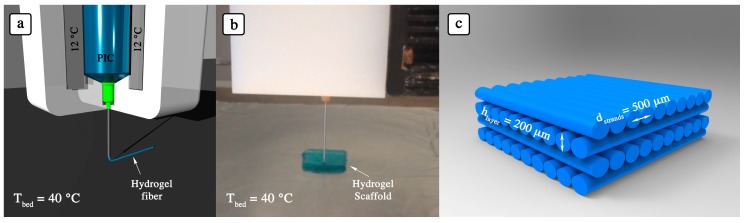
3D printing of PIC hydrogel. (**a**) Schematic representation of low-temperature cartridge of 3D-Bioplotter. (**b**) 3D printing of PIC hydrogel: the low-temperature cartridge containing the PIC hydrogel was kept at 12 °C while the temperature of the printing bed was set at 40 °C. (**c**) Schematic representation of the printed PIC hydrogel with 0–90° fiber orientation.

Thus, in our 3D printing experiments, we adjusted the cartridge temperature between 12 and 15 °C where PIC solution was extrudable and held stable rheological properties. Additionally, the bed temperature was set to 37–40 °C to attain both a stable gel after extrusion and printing conditions close to physiological temperature (Figure 2).

### 3.3. Adjusting 3D Printing Parameters for PIC Hydrogels

After optimizing the temperatures of the cartridge and bed, we performed a series of experiments to identify the best extrusion pressure and XY plotting speed for the 3D printing process. As an extrusion nozzle, we used a blunt metal needle (23 G, inner diameter of 330 µm, outer diameter of 650 µm, 3.81 cm long, general purpose dispersing), as it ensured proper strut formation. For instance, the use of smaller nozzle diameters resulted in recurring needle clogging, while larger diameters or shorter needles generally led to an excessive amount of hydrogel deposition.

**Figure 3:**
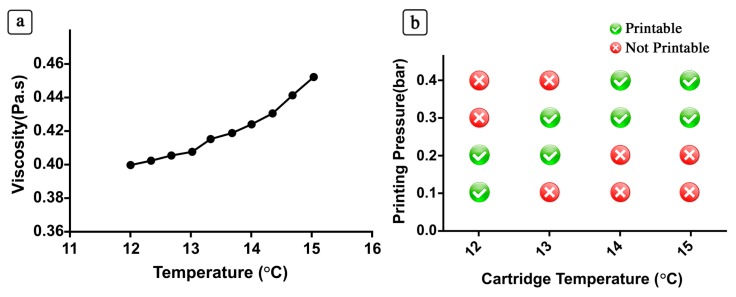
Viscosity and 3D printing parameters of the PIC hydrogels for a range of temperatures. In (**a**) the effect of temperature over the viscosity of a 5 mg/mL PIC solution is shown while in (**b**) the printability of PIC solution and the dependence of printing pressure on cartridge temperature are reported.

Thereafter, we studied the changes in the viscosity of the PIC solution within a range of temperatures (12–15 °C) and the relative pressure needed to extrude hydrogel struts. The obtained results are shown in Figure 3. As it can be noticed, the viscosity of the PIC solution at 12 °C was ~0.39 Pas, and 0.1 bar was sufficient to attain regular struts (Figure 3b). However, when the temperature was increased to 15 °C, we measured a 10% increase in viscosity, and the required pressure for strut deposition increased to 0.4 bar.

The printing pressure is a crucial parameter during 3D bioprinting experiments, as this may negatively affect the cell viability. For example, an excessive shear-stress may results in cell death due to the disruption of cell membrane [33]. The pressure values used in our study were significantly lower when compared to previous studies using different reverse thermoresponsive hydrogels [19,34]. For instance, Wu et al. used a pressure range of 3–18 bar for printing a 3D microvascular networks with 23% (*w*/*v*) Pluronic F127. Considering the >10-fold lower printing pressures, the PIC hydrogels exhibit superior performance when compared to the other reverse thermoresponsive hydrogels.

**Figure 4:**
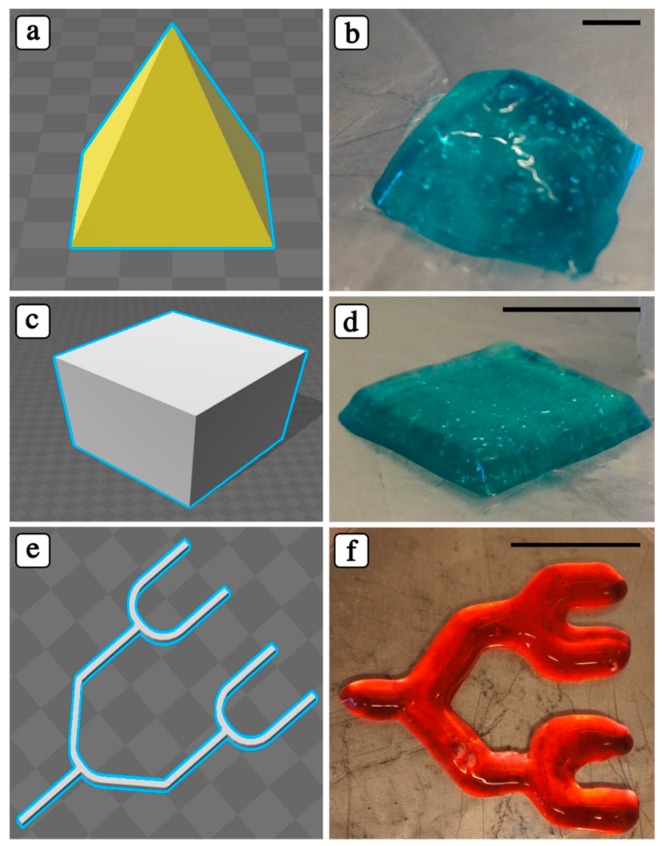
CAD and optical images of 3D-printed objects. All solids were printed with a layer thickness of 200 μm and a distance between fibers in the X–Y plane of 500 μm. Scale bars: 10 mm.

To demonstrate the possibility of 3D printing PIC hydrogels and the robustness of our deposition system, we printed a PIC solution to form different geometric structures (Figure 4). The printing speed in X-Y planes was altered according to the structure geometry, and the printing speed was determined as 30 ± 5 mm/s for cube, pyramid, and branched channels.

The layer thickness is directly linked to the way the consecutive, deposited layers bond, and how the 3D structure stacks up in z plane. The layer thickness must be set slightly lower than the strand thickness in order to attain adequate bonding between the layers. In theory, the layer thickness and the strand diameter should be equal—i.e., the cross-section of the strands could be considered as a perfect circle—at the optimized printing pressure and speed. Nevertheless, when working with hydrogel materials, most of the times an elliptical cross-section is attained [35]. Considering that the cross-section area should be constant at optimized pressure and speed, regardless of the shape of the cross-section, the relationship between layer thickness and the distance between the strands can be estimated theoretically. Given the range of pressure, printing temperatures and speed, the layer thickness was set to 200 µm and the distance between fibers was adjusted to 500 µm.

The printed scaffolds showed structural stability and endurance both during and after printing, when they were kept at room temperature or at 37 °C without any additional crosslinker and any further process. The simplicity of 3D printing of PIC hydrogels, their structural stability at 37 °C, the high water content of the hydrogel network (99.5% of the overall weight), their ECM-like characteristics, and their cytocompatibility [22,23,24,25] demonstrate the suitability of PIC hydrogels for 3D bioprinting.

As shown in Figure 4, a controlled inner microstructure cannot be attained, due to the low mechanical properties of the PIC hydrogels. However, such low mechanical properties may represent an advantage during cell culture, as they may better support cell migration and proliferation and nutrient and waste diffusion.

### 3.4. Creating Dual Hydrogel System: PIC as Fugitive Material

Besides fabricating self-standing 3D structures, fabricating microarchitectures that involves patterning is also an emerging field. In the case of using biocompatible hydrogels to create micro-patterns in a stable polymer, biocompatible hydrogels can be used as secondary hydrogel to create a dual hydrogel system. On the other hand, it is also possible to use patterned materials as fugitive material and remove them through a stimulus (typically thermal) to form the desired structures inside the stable polymer. In different studies, reverse thermoresponsive hydrogels have been used to attain either a co-culture system to create complex 3D constructs in different hydrogels, or as fugitive 3D structures [19,36,37]. Thus, we evaluated a potential application of PIC hydrogel for such systems.

**Figure 5:**
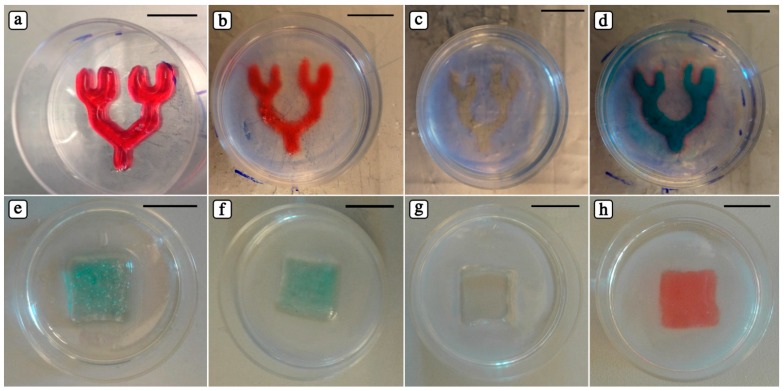
Optical images of 3D embedded constructs that are printed (**a**,**e**), covered with GelMA and photocrosslinked (**b**,**f**), evacuated (**c**,**g**), and perfused with a water-soluble food coloring (**d**,**h**) (scale bars: 10 mm).

To create our dual hydrogel systems, we used Gelatin MethAcrylate (GelMA) as outer and PIC as inner, or fugitive, hydrogel. GelMA has been previously studied for different biomedical applications thanks to its biocompatibility, ease of processing and capability of forming stable hydrogels at body temperature (37 °C) after UV photo-crosslinking [27,38]. The whole procedure is composed of four steps: (i) 3D PIC hydrogels are deposited (Figure 5a,e); (ii) warm GelMA solution (37 °C) is poured over the printed PIC structures and UV photo-crosslinked (Figure 5b,f); (iii) PIC hydrogel is removed by washing the overall constructs with cold PBS (4 °C) to open the channels rapidly (Figure 5c,g), and (iv) templated microchannels can be perfused (Figure 5d,h). Since the temperature of GelMA solution is above PIC gelation temperature, 3D printed PIC constructs can be finely preserved inside a GelMA network.

Thanks to its cytocompatibility [22], the role of PIC hydrogel within such dual hydrogel systems can be double: in fact, from one side, it can be used simply as a fugitive ink to template microchannels within the second gel, and, from the other side, it can be loaded with cells and act as a matrix supporting cell proliferation/differentiation. Furthermore, when compared to previously reported dual hydrogel systems based on Pluronic F127 (another synthetic polymer showing reverse thermal gelation for polymer concentration above 20% *w*/*v*), [19] PIC hydrogel offers the advantage of undergoing gelation with a polymer concentration 40 times lower. The high concentration of Pluronic F127 has been proved to be cytotoxic to multiple cells lines [18,39,40,41] and, as a consequence, Pluronic F127 has been used in dual hydrogel systems only as fugitive material [19]. Indeed, the high polymer concentration used in the case of Pluronic F127 allows to attain a higher printing resolution, but to the detriment of cell compatibility.

The ease of deposition and removal, the possibility to fabricate stable hydrogel constructs at 37 °C, as well as its high cytocompatibility make PIC hydrogels considerably preferable, paving the road for potential future applications.

## 4. Conclusions

In this study, we proposed a new synthetic biocompatible polymer for the formulation of bioinks which are suitable for the fabrication of 3D constructs and dual hydrogel systems via 3D printing. Its cytocompatibility, its unique reverse thermoresponsive properties, and its 3D printability make PIC polymer an interesting component of bioinks to be used to fabricate 3D matrices for cell studies. Furthermore, the capability of forming gels in 40-fold lower concentrations compared to other reverse thermoresponsive hydrogels, such as Pluronic F-127, represents a peculiar feature that, in the near future, will be further explored and optimized for dual cytocompatible hydrogel systems. Such a system, in fact, opens new routes for fundamental co-culture studies of cell niches, as dual hydrogel system can nurture and support different cell types, with different mechanical properties and different 3D architecture.

Finally, further improvements in PIC bioink formulation—for instance with the addition of colloidal particles to enhance its rheological behavior—may allow attaining increased pattern complexity and printing resolution. We believe that the proposed approach can be a valid system for the fabrication of functional and complex 3D tissues.
